# How Comprehensive Are Canadian Plastic Surgery Fellowship Websites?

**DOI:** 10.7759/cureus.15815

**Published:** 2021-06-21

**Authors:** Sahil Chawla, Jeffrey Ding, Sarim Faheem, Sandeep Shelly, Faisal Khosa

**Affiliations:** 1 Medicine, The University of British Columbia, Vancouver, CAN; 2 Faculty of Science, The University of British Columbia Okanagan, Kelowna, CAN; 3 Otolaryngology, Head and Neck Surgery, Emory University School of Medicine, Atlanta, USA; 4 Radiology, Vancouver General Hospital, Vancouver, CAN

**Keywords:** plastic surgery, program websites, fellowship programs, website comprehensiveness, online content

## Abstract

Background

Online fellowship program websites are more commonly becoming the primary information resource used by prospective applicants. This study aimed to analyze the online content of Canadian plastic surgery fellowship program websites.

Methods

The content of all accredited Canadian Plastic Surgery fellowship program websites was evaluated using a 75-point criterion in the following ten domains: recruitment, faculty, residents/fellows, research and education, surgical program, clinical work, benefits, and career planning, wellness, environment and gender of faculty leadership.

Results

On average, fellowship program websites obtained a score of 29.9 (SD=12.6). No correlation was detected between program websites and location (P > 0.05) nor by ranking (P > 0.05).

Conclusions

Most Canadian plastic surgery fellowship program websites lacked content relevant to prospective applicants. More comprehensive fellowship program websites may be of benefit to prospective applicants and the programs.

## Introduction

The Coronavirus Disease 2019 (COVID-19) pandemic has caused an unprecedented disruption in medical education [1-3]. For plastic surgery residents, mandates to flatten the curve came with an increase in online learning and virtual grand rounds [4]. The pandemic affected plastic surgery fellowship programs and prospective applicants preparing their fellowship applications.

Prior studies have shown that residents are more frequently turning to the internet during the fellowship application process [5,6]. Due to the restrictions on traveling and in-person interviews, the dependence on online information is expected to increase. Fellowship program websites provide important information, including location, accreditation status, salary, career incentives, and unique opportunities specific to the institution. The quality of program websites has been shown to affect applicants’ decisions on where to apply and their order of rank lists [5-8]. Therefore, website content may be prospective applicants’ primary information resource when preparing for application submissions.

To the best of our knowledge, prior research has not assessed Canadian plastic surgery fellowship program websites. The objective of this study was to evaluate the comprehensiveness of Canadian plastic surgery fellowship program websites using established criteria. In turn, we aimed to identify potential areas of development and assess for any correlations between website comprehensiveness and the programs’ geographic location and medical school ranking.

## Materials and methods

Data collection

Our methodology has been validated in several recent publications [9-11]. This study was exempt from institutional review board approval as the data was extracted in its entirety from publicly available resources. The Canadian Resident Matching Service (CaRMS) was searched to compile a list of medical schools that offered accredited plastic surgery fellowship training (n=12). These program websites were analyzed based on a previously validated 75-point criterion [9,12-14]. The criteria included the following 10 subcategories: recruitment, faculty, residents/fellows, research and education, surgical program, clinical work, benefits, and career planning, wellness, environment, and gender of leadership faculty. All programs had publicly available website data that were freely accessible. All data were[GP1] collected between July and August 2020. Each criterion was quantitatively analyzed by recording its presence or absence, and no assessment on information quality was made. No point was given if the information was present on a third-party website.

All fellowship programs were analyzed by location and ranking to assess for potential correlations with geography and medical school ranking. Programs were designated with 1 of the 6 Statistics Canada regions (Atlantic, Quebec, Ontario, Prairies, British Columbia, Territories) [15]. Canadian medical schools’ rankings were used [16], as no institution ranking according to fellowships currently exists. Medical schools were divided into 3 groups comprising the high, medium, and low-ranked medical schools. 

Statistical analysis

The statistical analysis was performed using Statistical Analysis Software 9.4 (SAS 9.4). The criteria per subcategory are shown in the results section. Total scores were calculated by summing up all the criteria under the fellowship program individually. The rank was described as high (1-5), medium (6-10), and low (>11). Total scores were also calculated for each subcategory. Analysis of Variance (ANOVA) procedure was performed to evaluate the difference in total mean criteria scores stratified by rank and geographic location. A p-value of 0.05 was considered significant.

## Results

The descriptive data for the criteria against which the program websites were evaluated are summarized in Table 1. On average, Canadian plastic surgery fellowship websites obtained a score of 29.9 (SD=12.6), indicating 39.9% completeness overall. The most frequent information included: contact email (100%), mailing address (100%), educational resources available (100%), program director name (83%), recruitment details description (75%), and meeting and conference opportunities (66.7%). The least common criteria found on the websites were: housing options (0%), work hours (0%) and incentives (0%), and salary (0%). For the location, the regional distribution is as follows: Quebec (3), Ontario (4), Prairies (3), Atlantic (1), and British Columbia (1). Out of 12 websites, five programs ranked high, four ranked medium, and the other four ranked low. ANOVA procedure did not find a statistically significant difference for total scores based on location (p-value = 0. 2518) and rank (p-value = 0. 7539) (Table 2). Although not significant, some discrepancies are present depending on the location of the program. However, the results should be interpreted with caution due to the small sample size per sublocation.

**Table 1:** Criteria examined in the websites *Abbreviations*: CaRMS (Canadian Resident Matching Service), n (number), IMG (International Medical Graduate), PGY (Postgraduate Year), CMA (Canadian Medical Association), and OMA (Ontario Medical Association)

Website Criteria	Fellowship (n = 12)
Recruitment	Websites with information, n (%)
Contact email address for the program	12 (100)
Mailing address	12 (100)
Selection criteria	7 (58.3)
Interview process	1 (8.3)
Interview dates	1 (8.3)
Electronic Application Service or CaRMS	9 (75)
Research requirements	8 (66.7)
Recruitment details	9 (75)
4th-year medical student electives	-
IMG information	8 (66.7)
Program Description	9 (75)
Program director name	10 (83.3)
New residents per year	1 (8.3)
Number of total Plastic residents/fellows	3 (25)
Number of total fellows (across all subspecialties)	0 (0)
Number of total staff/ attending (across all subspecialties)	3 (25)
Message from the program director	4 (33.3)
Message from the department chair	3 (25)
Sub-total	8.3 (2.5)
Faculty	
Comprehensive faculty listing (with names)	8 (66.7)
Specialty	9 (75)
Photos	9 (75)
Educational background	5 (41.7)
Research interests	6 (50)
Research publications	6 (50)
Awards	4 (33.3)
Research presentations	3 (25)
Sub-total	4.1 (2.8)
Residents/Fellows	
List of current Fellows (with names)	5 (41.7)
Fellow year status (ex. PGY)	4 (33.3)
Individual or group photo	5 (41.7)
Past Alumni names	4 (33.3)
Alumni locations/ where they work now	1 (8.3)
Sub-total	1.6 (1.4)
Research and Education	
Research Opportunities	7 (58.3)
Current research projects	7 (58.3)
Past research projects	6 (50)
Grants awarded	5 (41.6)
Journal club	8 (66.7)
Meetings and conference opportunities	8 (66.7)
Teaching	6 (50)
Grand round conferences	8 (66.7)
Educational resources available to fellows	12 (100)
Research requirements	8 (66.7)
Sub-total	6.2 (2.7)
Surgical Program	
Responsibility progression	5 (41.7)
Call requirements (Night Float)	2 (16.7)
Surgical case	3 (25)
Surgical statistics	2 (16.7)
Imaging case numbers	2 (16.7)
Imaging equipment description	4 (33.3)
Ultrasound Component	3 (25)
Simulation	4 (33.3)
Robotics	3 (25)
International opportunities	9 (75)
Sub-total	3.1 (2.7)
Clinical Work	
Expected case load	3 (25)
Rotation schedule	6 (50)
Work hours	0 (0)
Call Schedule	2 (16.7)
On-call responsibilities	2 (16.7)
Evaluation	3 (25)
Sub-total	1.3 (1.5)
Benefits and career planning	
Incentives	0 (0)
Salary	0 (0)
Vacation	3 (25)
Maternal leave mentioned	3 (25)
Paternal leave mentioned	3 (25)
Moonlighting mentioned	1 (8.3)
Career placement	1 (8.3)
Future study opportunities	5 (41.7)
Sub-total	1.3 (1.6)
Wellness	
Resident wellness	3 (25)
Associations with professional organizations (OMA, CMA, etc.)	3 (25)
Harassment Policy	6 (50)
Sub-total	1 (1.2)
Environment	
Hospitals	4 (33.3)
Neighborhood information	0 (0)
Local attractions	0 (0)
Social events	5 (41.7)
Pictures of social events	8 (66.7)
House options	0 (0)
Sub-total	1.4 (1.2)
Gender	
Gender of Department chair	8 (66.7)
Gender of program director	9 (75)
Sub-total	1.4 (0.67)
Grand Score	29.9 (12.6)

**Table 2:** Mean and SD for number of criteria met, stratified by location and rank *Abbreviations: *SD (Standard Deviation), NA (Not Applicable)

Characteristic	Fellowship Mean (SD)
Location	
Atlantic	31.0 (NA)
British Columbia	50.0 (NA)
Ontario	35.0 (10.6)
Prairies	19.7 (6.1)
Quebec	26.3 (15.4)
Rank	
High	33.75 (16.0)
Medium	29.5 (11.8)
Low	26.5 (12.2)

## Discussion

Given that almost half of all plastic surgery residents pursue subspecialty fellowship training [17], it is an invaluable opportunity to hone one’s skills and improve job prospects [18]. As the internet’s popularity for sharing information with medical professionals continues to increase, it is important that medical professionals follow suit. Plastic surgery fellowship directors consider factors like recommendation letters, interview performance, and professionalism when selecting a competitive applicant [19-21]. However, by creating complete and comprehensive program websites, fellowship programs could attract more competitive applicants. Our study results show that currently, Canadian plastic surgery fellowship program websites are underutilized. This is in keeping with results from prior studies evaluating other medical specialties’ websites [9,22,23].

Of the 12 Canadian plastic surgery fellowship websites, only three mentioned paternal leave. These findings may have negative consequences for fellowship programs and prospective fellows. Despite the majority of physicians’ residency and fellowship training coinciding with childbearing age [24], variable institutional support exists. Paid parental leave during fellowship training has the potential to decrease postpartum maternal depression and intimate partner violence while improving child development and the mother’s physical recovery [25]. It is important to consider that while parental leave policies are the first step, a supportive program culture that encourages participation in these policies is also a necessary ingredient [26]. Adopting and marketing policies that increase access to paid parental leave during training has the potential to recruit competitive applicants, ultimately benefitting both the program and trainee.

While almost one-half of all identified programs described rotation schedules, only half of those described the expected caseload, and none described work hours or salary. This is similar to other plastic surgery fellowships [20,27,28]. Providing prospective applicants with few details on the expected workload may result in the difference between applying or not. This potential discrepancy in expectations between the program and successful applicants may contribute to the growing issue of physician burnout.

This study demonstrates that a considerable amount of information is missing from the majority of fellowship programs' websites. Some reasons for this current state of missed fundamental information on websites include the presumption that information is not important and therefore not explicitly stated. Another possibility is a lack of recognition of the changing trends for obtaining information on fellowships. Programs may assume that most applicants are getting their information by word of mouth. Programs may also outsource website maintenance to third-party companies, which may not consult physicians regularly. Additionally, given a large number of qualified applicants, fellowship programs may perceive little incentive to develop more comprehensive program websites. Interestingly, a small portion of plastic surgery sub-specialty programs do not have enough fellows for all of their seats [27]. Therefore, more comprehensive websites may help to recruit future applicants. 

Although not significant, fellowship programs within the Prairie region did have a considerably fewer number of items compared to Western Canada (19.7 vs. 50.0, P = 0.0099) (Table 2); however, this should be interpreted with caution as Western Canada only has a single program (n = 1), compared to three within Prairies. Medical school rankings of low, medium or high did not have a statistically significant difference in the content of program websites; however, there is a step-wise increase in information on program websites going from low to high ranking. This may be a logical finding as higher-ranking medical schools may have higher-ranking fellowship programs and more comprehensive program websites. This finding is likely multifactorial and may represent a growing awareness among all universities to maintain an up-to-date and informative online resource for prospective applicants.

Overall, the usage of Canadian plastic surgery fellowship program websites during the application cycle remains uninvestigated. Further research should survey plastic surgery fellowship trainees to investigate what program characteristics influenced their decision to apply and also where they received most information about their program from. This study could be used further by the Canadian Society of Plastic Surgery (CSPS) to create a centralized administrative process that mandates the comprehensiveness of program websites and ensures they are kept up to date.

Limitations

This study has several limitations. Firstly, fellowships often draw international applicants and are a less standardized process. Fellowship spots wax and wane in volume, and due to the limited number of plastic surgery fellowship programs in Canada, results should be interpreted with caution. Secondly, only the information available on the program’s website was included, so it is possible that each program’s information is available online; for example, via premed forums, email chains, or word of mouth. Thirdly, little literature is present that shows what website content is most appreciated by fellows. Lastly, this data collection was completed in August 2020, and it is possible that plastic surgery fellowship program websites have been since updated.

## Conclusions

Given the travel restrictions and virtual interviews due to COVID-19, it is more important than ever that fellowship program websites are kept up to date so that applicants may be equipped with the necessary information prior to applying. Canadian plastic surgery fellowship program websites are lacking in comprehensiveness pertinent to prospective applicants. Future studies should assess which factors were important to plastic surgery fellows when choosing to apply and which program website components fellows found most useful. It is also important to assess if/how fellowship program websites reflect curriculum change brought about by the COVID-19 pandemic.
